# Altered habenular connectivity in chronic low back pain: An fMRI and machine learning study

**DOI:** 10.1002/hbm.26389

**Published:** 2023-06-12

**Authors:** Cui Ping Mao, Yue Wu, Hua Juan Yang, Jie Qin, Qi Chun Song, Bo Zhang, Xiao Qian Zhou, Liang Zhang, Hong Hong Sun

**Affiliations:** ^1^ Department of Medical Imaging Second Affiliated Hospital of Xi'an Jiaotong University Xi'an Shaanxi China; ^2^ School of Computer Science and Engineering Xidian University Xi'an Shaanxi China

**Keywords:** chronic low back pain, dynamic causal modelling, habenula, resting‐state functional connectivity, support vector machine

## Abstract

The habenula has been implicated in the pathogenesis of pain and analgesia, while evidence concerning its function in chronic low back pain (cLBP) is sparse. This study aims to investigate the resting‐state functional connectivity (rsFC) and effective connectivity of the habenula in 52 patients with cLBP and 52 healthy controls (HCs) and assess the feasibility of distinguishing cLBP from HCs based on connectivity by machine learning methods. Our results indicated significantly enhanced rsFC of the habenula‐left superior frontal cortex (SFC), habenula‐right thalamus, and habenula‐bilateral insular pathways as well as decreased rsFC of the habenula‐pons pathway in cLBP patients compared to HCs. Dynamic causal modelling revealed significantly enhanced effective connectivity from the right thalamus to right habenula in cLBP patients compared with HCs. RsFC of the habenula‐SFC was positively correlated with pain intensities and Hamilton Depression scores in the cLBP group. RsFC of the habenula‐right insula was negatively correlated with pain duration in the cLBP group. Additionally, the combination of the rsFC of the habenula‐SFC, habenula‐thalamus, and habenula‐pons pathways could reliably distinguish cLBP patients from HCs with an accuracy of 75.9% by support vector machine, which was validated in an independent cohort (*N* = 68, accuracy = 68.8%, *p* = .001). Linear regression and random forest could also distinguish cLBP and HCs in the independent cohort (accuracy = 73.9 and 55.9%, respectively). Overall, these findings provide evidence that cLBP may be associated with abnormal rsFC and effective connectivity of the habenula, and highlight the promise of machine learning in chronic pain discrimination.

## Graphical abstract

Our study revealed abnormal resting‐state functional connectivity and effective connectivity of the habenula with cortical/subcortical areas in patients with chronic low back pain than healthy control subjects, and the habenula connectivity can be used to distinguish chronic low back pain and healthy controls reliably by machine learning methods.

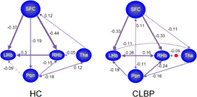

## 1 INTRODUCTION

Low back pain (LBP) is a leading cause of years lived with disability globally (James et al., 2018). Recurrences of LBP are common, and some patients end up with persistent disabling pain (Hartvigsen et al., 2018). Numerous studies have regarded chronic LBP (cLBP) as a disorder affected by a range of biophysical, psychological and social factors (Hartvigsen et al., 2018; Ho et al., 2022). In previous studies, structural and functional brain abnormalities were repeatedly reported in patients with cLBP (Kregel et al., 2015; Tu et al., 2019; Tu et al., 2020). Despite significant progress in research, the mechanisms underlying cLBP have yet to be fully elucidated due to the nonspecific nature of the majority of cLBPs (Maher et al., 2017). Recently, convergent preclinical data have implicated the habenula in pain modulation and analgesia (Cohen & Melzack, 1993; Shelton, Becerra, & Borsook, 2012); however, its function in cLBP remains to be clarified.

The habenula is a bilateral epithalamic structure linking the forebrain to midbrain regions, and it is composed of a medial and lateral subdivision (Namboodiri et al., 2016). In the past few decades, numerous studies have implicated the habenula in the pathogenesis of neuropsychiatric disorders (Cui et al., 2018; Lee & Goto, 2021; Wu et al., 2020). Only a small number of investigations have implicated the putative role of the habenula in pain modulation and analgesia (Du et al., 2021; McIlwrath et al., 2020). For example, acute pain can be attenuated by facilitating endocannabinoid signalling in the lateral habenula in rats (Fu et al., 2021). The lateral habenula can serve as a potential therapeutic target for neuropathic pain in mice (Du et al., 2021) or rats (Khalilzadeh & Saiah, 2017). Although the habenula has been shown to be crucial in pain modulation (mainly acute pain) in animals, its role in chronic pain, especially in human conditions, has yet to be determined. Evidence concerning the link between the habenula and cLBP remains scarce.

To date, resting‐state functional magnetic resonance imaging (fMRI) (Fox & Greicius, 2010) has been used to identify the intrinsic connectivity of the brain in patients with cLBP (Ayoub et al., 2019; Letzen et al., 2020). Based on fMRI, we can measure resting‐state functional connectivity (rsFC) (K. J. Friston, 2011; K. J. Friston et al., 2003) and effective connectivity reflecting nondirectional/directional statistical dependencies between brain regions (K. J. Friston, 2011). Dynamic causal modelling (DCM) has been widely used to characterize effective connectivity (K. J. Friston et al., 2014). In addition, machine learning has been increasingly used in pain research (D'Antoni et al., 2022; Matsangidou et al., 2021) to automatically identify brain signatures of patients and make accurate classifications (Sarker, 2021). In this study, resting‐state fMRI and machine learning were both used to explore the habenular connectivity patterns in cLBP.

In this study, we hypothesized that cLBP would be associated with altered rsFC and effective connectivity of the habenula, which would be correlated with clinical measures. We also assessed the feasibility of distinguishing cLBP from healthy controls (HCs) by machine learning methods.

## 2 METHODS

### 2.1 Participants and clinical measures

Fifty‐two patients with cLBP (eighteen females, 47.1 ± 12.6 years of age) were recruited from the Outpatient Clinic, Department of Orthopaedics, in the Second Affiliated Hospital of Xi'an Jiaotong University. Fifty‐two HCs (eighteen females, 48.8 ± 10.1 years of age) were recruited via advertisement. The study was approved by the Research Ethics Committee of the Second Affiliated Hospital of Xi'an Jiaotong University (number 2015049). The diagnosis of cLBP was performed by two experienced clinicians (Q. J. and S. QC). The HCs were recruited from the surrounding area through advertisements and word‐of‐mouth. The HCs were required to have no history of any pain or neurological disorders and met the following exclusion criteria. All participants provided written informed consent prior to entering the study. The medications for all cLBP patients can be found in the Supplemental materials.

#### 2.1.1 Inclusion criteria

Participants were considered to be in the cLBP group if they (1) met the criteria of the International Association for the Study of Pain for the diagnosis of cLBP (Merskey & Bogduk, 1994; Treede et al., 2019); (2) were 18–65 years of age; (3) had chronic pain primarily localized to the lumbosacral region, with or without pain radiating to the buttocks, thighs, or legs; and (4) had persistent pain for more than half a year. The source of pain was not distinguished.

#### 2.1.2 Exclusion criteria

Participants were excluded if they (1) had concomitant psychiatric disorders, substance abuse/dependence, treatment with antidepressants or other medications that would affect the study results; (2) had a history of significant head trauma; (3) had chronic pain in other body sites; or (4) had contraindications to MR imaging (i.e., metal implants or severe claustrophobia).

#### 2.1.3 Questionnaires

The short‐form McGill Pain Questionnaire (Melzack, 1987) was used to assess the intensity of pain, in which the subjects rated the intensity of pain on a visual analogue scale (VAS) from 0 to 10 (0 = “no pain,” 10 = “highest pain imaginable”) on the day of the scan. The Hamilton Depression (HAMD) scale (Williams, 1988) was used to evaluate the psychological symptoms of all participants.

### 2.2 MRI acquisition

MRI data were acquired on a 3.0‐T whole‐body MR scanner (Signa HDXT, GE Healthcare) equipped with a standard eight‐channel head coil. All participants were required to abstain from medications for 12 h before the MR scan. High‐resolution anatomic T1‐weighted images were acquired using a 3D‐T1‐weighted fast spoiled gradient echo sequence with the following parameters: time to repetition (TR): 10.8 ms, time to echo (TE): 4.8 ms, flip angle: 15°, slice thickness = 1 mm, space between slices = 0, voxel size: 1 × 1 × 1 mm^3^, field of view: 256 mm × 256 mm, 150 slices. The resting‐state blood oxygen level‐dependent (BOLD) data were obtained using a gradient echo‐planar MR imaging sequence with the following parameters: TR: 2500 ms, TE: 30 ms, flip angle: 90°, slice thickness = 3 mm, zero gap, voxel size: 4 × 4 × 3 mm^3^, field of view: 256 mm × 256 mm, 50 slices, 158 volumes, lasting for 6 min 35 s.

### 2.3 FMRI data analysis

#### 2.3.1 MRI data preprocessing

The CONN toolbox version 20.b (Whitfield‐Gabrieli & Nieto‐Castanon, 2012) was used to perform the MRI data preprocessing. First, the first four volumes of the functional MRI data were discarded due to transient signal changes before magnetization reached a steady state. The preprocessing of each subject's individual MR scan was performed using a pipeline in the CONN toolbox, including functional realignment and unwarping, functional slice‐timing correction, structural segmentation and normalization, coregistration with the structural data (resampled target resolution for functional images = 2 mm isotropic voxels), functional normalization, functional outlier detection (ART‐based scrubbing, https://www.nitrc.org/projects/artifact_detect), and functional smoothing (full‐width‐at‐half maximum [FWHM] = 4 mm, used only for the rsFC analysis). A combination of aComCor (white matter and cerebral spinal fluid regions of interest [ROIs], 5 components each), scrubbing, motion regression (12 regressors: 6 motion parameters + 6 first‐order temporal derivatives), and filtering were used to reduce the noise (band: 0.008–0.09 Hz). The “outliers” refer to the regressors used in the scrubbing regression, that is, the images whose composite movement from a preceding image exceeded 0.5 mm, or if the global mean intensity was >3 standard deviations from the mean image intensity of the entire resting scan. Moreover, we also included the maximum interscan movement (framewise displacement) (Jenkinson et al., 2002) as a covariate in all group‐level analyses to reduce the effect of motion artefacts in cLBP patients and HCs.

#### 2.3.2 RsFC analysis of the habenula

**FIGURE 1:**
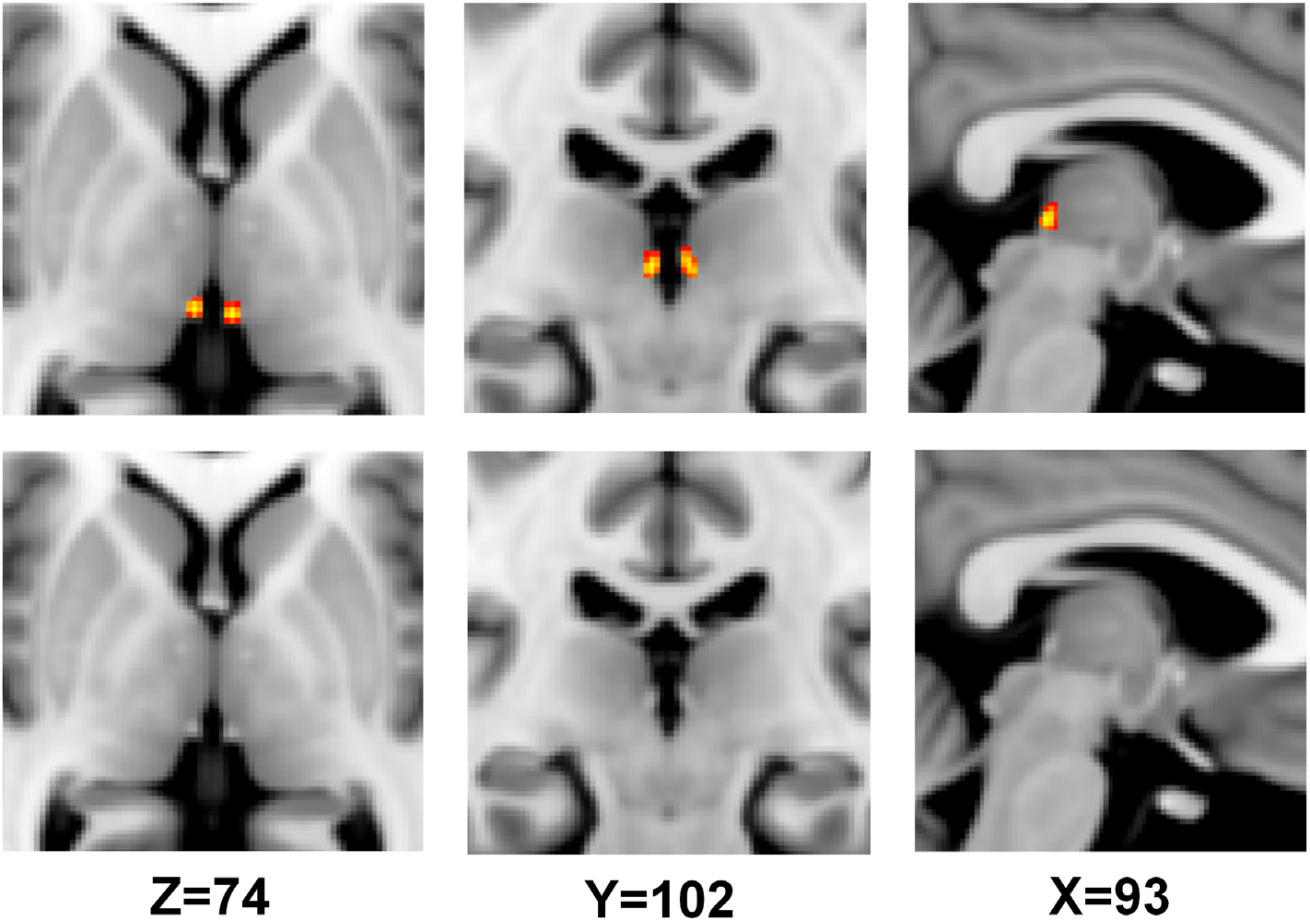
Location of the habenula in the brain. The left and right habenulas are adjacent to the medial dorsal nucleus of the thalamus. The red‐yellow part in the upper row is corresponding to the grey‐white ones in the lower row. The brighter regions represent the larger possibility of the habenula.

Seed‐based rsFC analysis was performed in the CONN toolbox. The seeds for the left and right habenula proposed by Kim et al. (2018) were combined into one seed as done in previous studies (Ely et al., 2016; Torrisi et al., 2017) (Figure 1) and in our previous publication (Mao et al., 2020). This approach aims to improve the detection power in the functional connectivity analyses of the habenula given its smaller size. Because the habenula is a tiny structure, any small changes of the placement of the seed can have a substantial effect on the rsFC results. We therefore checked and redefined the habenula seed. Firstly, we thresholded the habenula ROI and yielded a binary habenula mask including only voxels weighted as containing 50–100% habenula tissue. Second, FSL‐FNIRT was used to register the mask into standard Montreal Neurological Institute (MNI) space to make an ultimate mask (Figure 1) which would be used in the following rsFC analysis. The first‐level functional connectivity analysis yielded Fisher's r‐to‐z transformed bivariate correlations between the habenula and other voxels of the brain. Then, second‐level analyses were performed to determine group‐based differences in rsFC of the habenula and the whole brain after correcting for the effects of age, gender and HAMD scores. The results were considered significant at a threshold of voxelwise *p* < .005 uncorrected or cluster‐level *p* < .05 false discovery rate (FDR) corrected for the between‐group comparisons.

#### 2.3.3 RsFC analysis of a control seed

In addition to the habenula seed, we created a control seed containing spherical ROIs of bilateral thalamus (radius = 3 mm, left: *x* = −14, *y* = −24, *z* = 2; right: *x* = 14, *y* = −24, *z* = 2; Figure S1). We repeated the rsFC analysis using the thalamic seeds and compared the results with those obtained using the habenula.

#### 2.3.4 Effective connectivity analysis

DCM 12 (version 7771) (K. J. Friston, 2011; K. J. Friston et al., 2014) implemented in SPM12 was used to assess the effective connectivity of the habenula. The ROIs used in DCM analysis were the brain regions showing significant between‐group differences in the functional connectivity analyses by the CONN toolbox. Together, five ROIs were selected, including the left superior frontal cortex (SFC, *x* = −14, *y* = 10, *z* = 58); right thalamus (*x* = 14, *y* = 4, *z* = 6); left habenula (*x* = −3, *y* = −24, *z* = 2); right habenula (*x* = 5, *y* = −24, *z* = 2); and pons (*x* = 8, *y* = −20, *z* = −30). In square brackets, there are corresponding MNI coordinates of the centres of regions. The time series were extracted from the upper ROIs based on the preprocessed but unsmoothed data from CONN analyses. Seven models were specified, including a fully connected model, three models excluding one of the three regions including the SFC, thalamus, or pons, and three models excluding any two of the upper three regions. Fixed effects Bayesian model selection (BMS) (Stephan et al., 2009) was performed to determine the best model that balances the fit of the data and the model complexity. Then, Bayesian model averaging (Penny et al., 2010) was conducted to analyse the connectivity parameters, which were compared later. Finally, we only reported abnormal effective connectivity of the pathways from the habenula or to the habenula for between‐group comparisons. The effects will be reported if they have a posterior probability >.95.

Finally, to assess whether different connectivity methods (rsFC, effective connectivity) reflect similar neural interactions in the same subjects, we explored the relationship between the rsFC and effective connectivity of the habenula by Spearman rank correlation analysis as reported in previous studies (Paul et al., 2021; Rehme et al., 2013). The significance was assessed by performing a one‐sample *t* test (*p* < .05, FDR‐corrected for multiple comparisons).

### 2.4 Comparisons of habenular rsFC/effective connectivity in an independent cohort

To validate the results from the CONN and DCM analyses, we repeated these two analyses in an independent cohort including 34 cLBP patients and 34 HCs. The MRI data were obtained from the OpenPain Project database (https://www.openpain.org).

### 2.5 Machine learning

Support vector machine (SVM) learning was used to classify cLBP subjects and HCs implemented on the LIBSVM toolbox version 3.18 in MATLAB R2021a (Chang & Lin, 2011). The brain areas showing significant between‐group differences in the first dataset (52 HCs and 52 cLBP patients) were selected as ROIs, and the habenular rsFC was extracted centred at the coordinates (2‐mm radius sphere) for both datasets and used in the classification by SVM learning. All subjects were randomly split into a training set and a testing set 10 times. The number of cases in the training set and testing set were consistent (104 cases for the training set, 68 cases for the testing set). Tenfold cross‐validation was applied. To quantify the performance of the SVM classifier, classification accuracy, sensitivity, specificity, precision, and area under the curve were calculated. Additionally, the accuracy of the testing set was assessed by a permutation test with 1000 epochs as used in previous studies (Golland & Fischl, 2003; Mao et al., 2020). To determine the generality of the habenular rsFC as a discriminating factor in other classifiers, we compared the classification performance among SVM, linear regression (Pouromran et al., 2021) and random forest (Miettinen et al., 2021).

### 2.6 Statistical analysis

SPSS 24.0 (SPSS Inc, Chicago, IL) was used to perform the statistical analyses that were not included in the CONN and DCM analyses. Age and HAMD scores were compared by independent sample *t* tests or Mann–Whitney *U* tests based on the normality of the data and the homogeneity of variances. Gender differences were assessed by the chi‐squared test. Stepwise linear regression analysis was performed to explore the association between the rsFC/effective connectivity of the habenula showing significant between‐group differences and clinical measures (VAS scores, disease duration, and depression scores) in the cLBP group.

### 2.7 RsFC of the habenula with unsmoothed fMRI data

Habenula is a small structure. In order to keep the spatial accuracy and avoid the effect of neighbouring nuclei on results of the habenular rsFC as suggested in previous study (Mills et al., 2018), we repeated the rsFC analysis in both dataset by removing the smooth step in the preprocessing of the resting‐state fMRI data. The results from unsmoothed data were provided and compared with those obtained from smoothed data.

## 3 RESULTS

### 3.1 Demographics and clinical measures of all participants

There were no significant differences in age or gender between cLBP patients and HCs. The depression scores were significantly higher in cLBP patients than in HCs (*p* < .001). Detailed demographic and clinical characteristics are shown in Table 1.

**TABLE 1:** Demographical and clinical data.

Item	HC	CLBP	*p*
Number of all subjects	52	52	
Number of females (%)	34 (65%)	34 (65%)	.574
Age (years)	47.1 ± 12.6^a^	48.8 ± 10.1	.44
Age range (years)	24–65	28–65	
HAMD	0.6 ± 1.4	5.1 ± 5.4	<.001
VAS score		4.7 ± 1.8	NA
Disease duration (years)		7.8 ± 7.9 (0.5–30)	NA

Abbreviations: CLBP, chronic low back pain; HAMD, Hamilton Depression scale; HC, healthy control; NA, not available; VAS, visual analogue scale.

^a^ Mean ± standard deviation.

### 3.2 RsFC of the habenula

**FIGURE 2:**
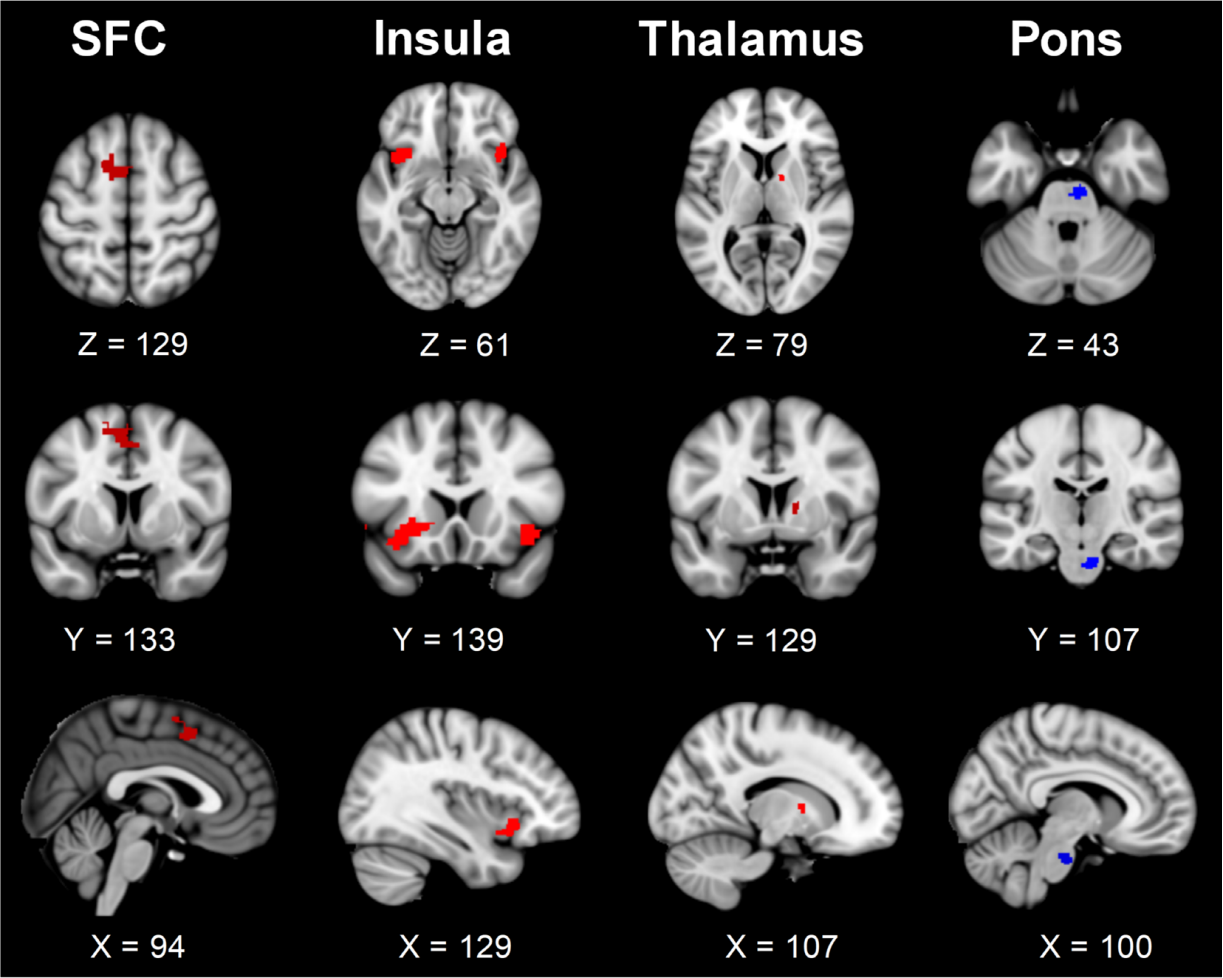
Resting‐state functional connectivity of the habenula. There was significantly enhanced (red) and decreased (blue) resting‐state functional connectivity of the habenula in patients with chronic low back pain compared to healthy controls. The connectivity values of the habenula with the thalamus and pons were obtained from small‐volume correction analyses. SFC, superior frontal cortex

Compared with HCs, patients with cLBP showed significantly enhanced rsFC between the habenula and several cortical/subcortical regions, including the left SFC (*p* = .013, FDR corrected), right thalamus (*p* = .022, FDR corrected), and bilateral insular cortex (left: *p* = .01, FDR corrected; right: *p* = .017, FDR corrected; Figure 2; Table 2). In addition, patients with cLBP also showed significantly decreased rsFC between the habenula and pons compared with HCs (*p* = .009, FDR corrected; Figure 2; Table 2).

**TABLE 2:** Group differences in the resting‐state functional connectivity of the habenula.

Group difference	Target area	Volume data			Voxels	*Z* value	*p*
		*X*	*Y*	*Z*			
HC < CLBP	Left SFC	−14	10	58	242	3.43	.013
	Right thalamus	14	4	6	16	3.42	.022
	Left insular cortex	−38	14	−12	335	4.00	.01
	Right insular cortex	42	18	−10	154	3.66	.017
HC > CLBP	Pons	8	−20	−30	53	4.88	.009

*Note*: The *p* values are corrected for false discovery rate.

Abbreviations: CLBP, chronic low back pain; HC, healthy control; SFC, superior frontal cortex.

### 3.3 RsFC of the thalamus

Compared with HCs, patients with cLBP showed significant decreased rsFC between thalamus and right precentral/parietal cortex (*p* < .001, FDR corrected) as well as cingulate cortex (*p* = .01, FDR corrected). In addition, patients with cLBP showed significantly enhanced rsFC between thalamus and left posterior insular cortex (*p* < .01, FDR corrected). The results can be seen in Supplemental Materials (Figure S2, Table S1).

### 3.4 Effective connectivity of the habenula

**FIGURE 3:**
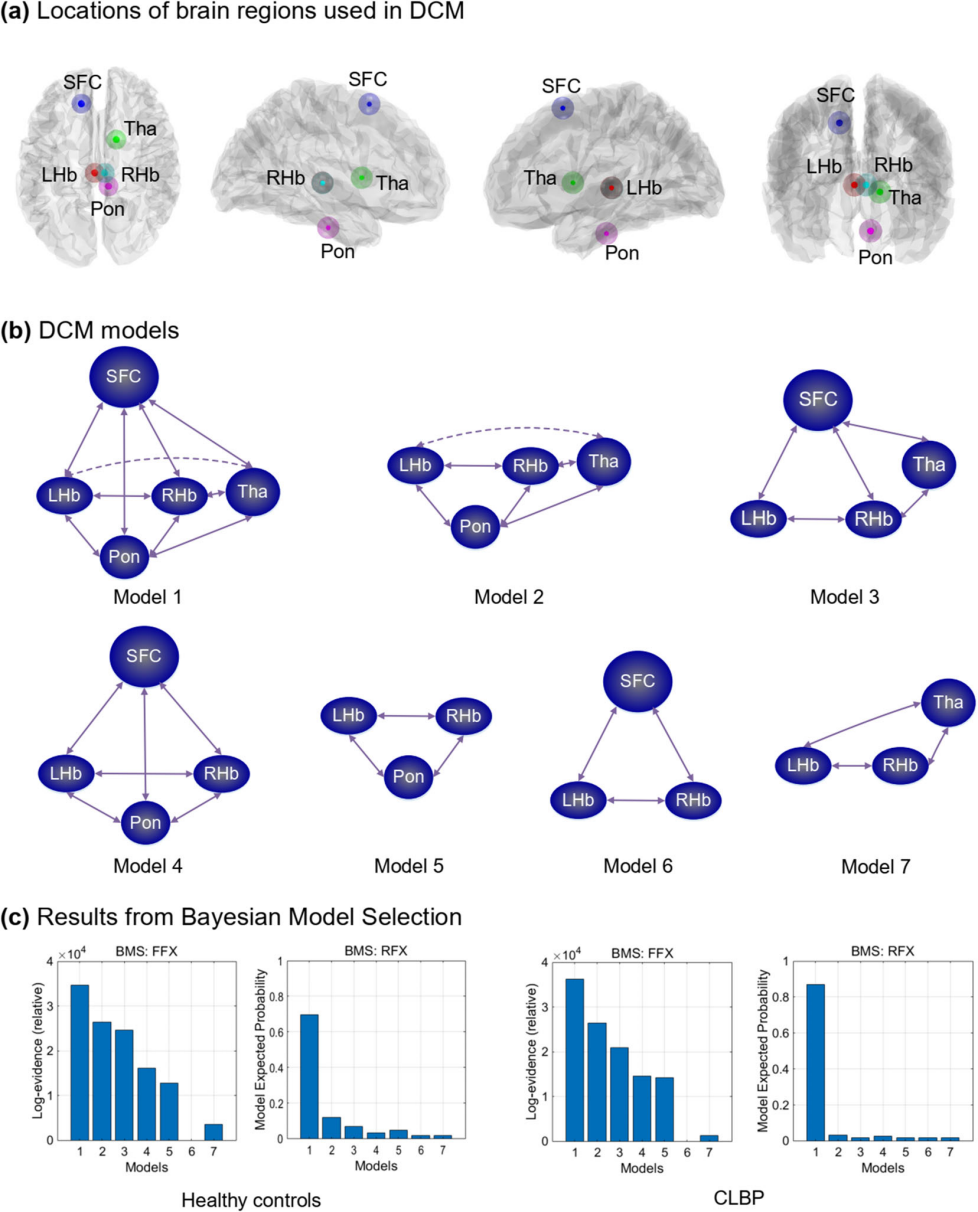
Effective connectivity of the habenula. (a) Locations of brain regions used in the dynamic causal modelling. (b) Seven models used in the DCM analysis. (c) Results from Bayesian model selection. BMS, Bayesian model selection; CLBP, chronic low back pain; DCM, dynamic causal modelling; FFX, fixed effect; LHb, left habenula; pon, pons; RFX, random effect; RHb, right habenula; SFC, superior prefrontal cortex; Tha, thalamus

**FIGURE 4:**
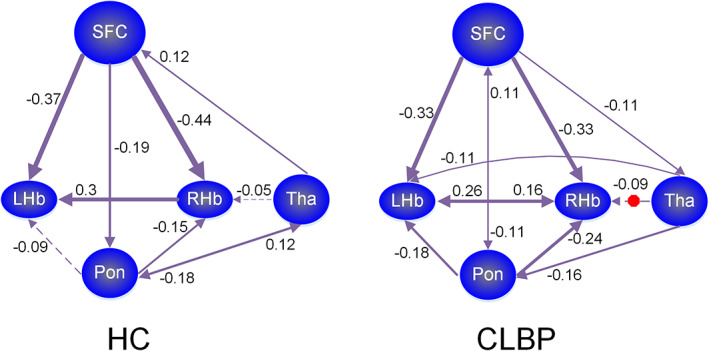
Winning models in dynamic causal modelling (DCM) at the group level. The numbers represent the connectivity parameters (Hz) of the winning model in the patients with chronic low back pain and healthy controls. The solid lines and dotted lines represent connectivity values greater/less than 0.1 Hz, respectively. The red octagons represent significant between‐group differences in that pathway. CLBP, chronic low back pain; HC, healthy controls; LHb, left habenula; pon, pons; RHb, right habenula; SFC, superior prefrontal cortex; Tha, thalamus

The locations of the brain regions used to extract time series in DCM analyses are shown in Figure 3a. Seven models used in DCM are shown in Figure 3b. The results from BMS revealed that the fully connected model was the best model in both the cLBP and HC groups (Figure 3c). It was the best model for 40 of 52 HCs and for 50 of 52 cLBP patients. For HCs, models 2–5 were the best models for six, three, one, and two subjects, respectively. For patients with cLBP, models 2 and 4 were the best models for one subject, respectively. The effective connectivity values of the habenula in the cLBP and HC groups are shown in Table 3. Between‐group comparisons indicated significantly enhanced effective connectivity of the right thalamus‐to‐right habenula pathway in cLBP patients (*p* = .012, FDR corrected) compared with HCs (Figure 4).

**TABLE 3:** Mean connection strengths (in Hz) of the habenula and other brain regions in healthy controls and patients with chronic low back pain.

Group	BMS	From SFC	From Tha	From LHb	From RHb	From Pons
HC	To SFC	0	0.056	0.035	0.057	0.037
	To Tha	−0.013	0	0.023	0.0002	0.115**
	To LHb	−0.37**	0.059	0	0.299**	−0.091
	To RHb	−0.437**	−0.05	0.104	0	−0.153
	To Pons	−0.185**	−0.181**	−0.007	−0.018	0
CLBP	To SFC	0	0.016	0.046	0.033	0.106**
	To Tha	−0.114**	0	0.032	−0.004	0.014
	To LHb	−0.326**	−0.105	0	0.257**	−0.178*
	To RHb	−0.329**	−0.092△	0.16**	0	−0.244**
	To Pons	−0.106*	−0.156**	−0.01	−0.018	0

*Note*: There are source regions in rows and target regions in columns. The * represents the parameters that have significantly nonzero values revealed from single group *t* test. The “△” represents significant between‐group differences of the habenular effective connectivity.

Abbreviations: BMS, Bayesian model selection; CLBP, chronic low back pain; HC, healthy controls; LHb, left habenula; RHb, right habenula; SFC, superior frontal cortex; Tha, thalamus.

* *p* < .05.

** *p* < .01.

### 3.5 RsFC and effective connectivity of the habenula in the independent cohort

The demographic data of the independent cohort are shown in Table S2. Between‐group comparisons suggested significantly enhanced rsFC between the habenula and right medial prefrontal cortex (MPF) (*p* = .003, FDR corrected), right thalamus (*p* = .015, FDR corrected), and left temporal cortex (*p* < 0.001, FDR corrected, Table S3) in patients with cLBP compared with HCs. Patients with cLBP also showed significantly decreased rsFC between the habenula and pons compared with HCs (Table S3). Second, between‐group comparisons suggested a significantly enhanced effective connectivity of the pons‐to‐right habenula pathway (*p* = .047, FDR corrected) in patients with cLBP compared to HCs after controlling for the effects of age, gender, and depression scores (Beck Depression Inventory, Table S4).

### 3.6 Classification of cLBP and HCs by machine learning

**FIGURE 5:**
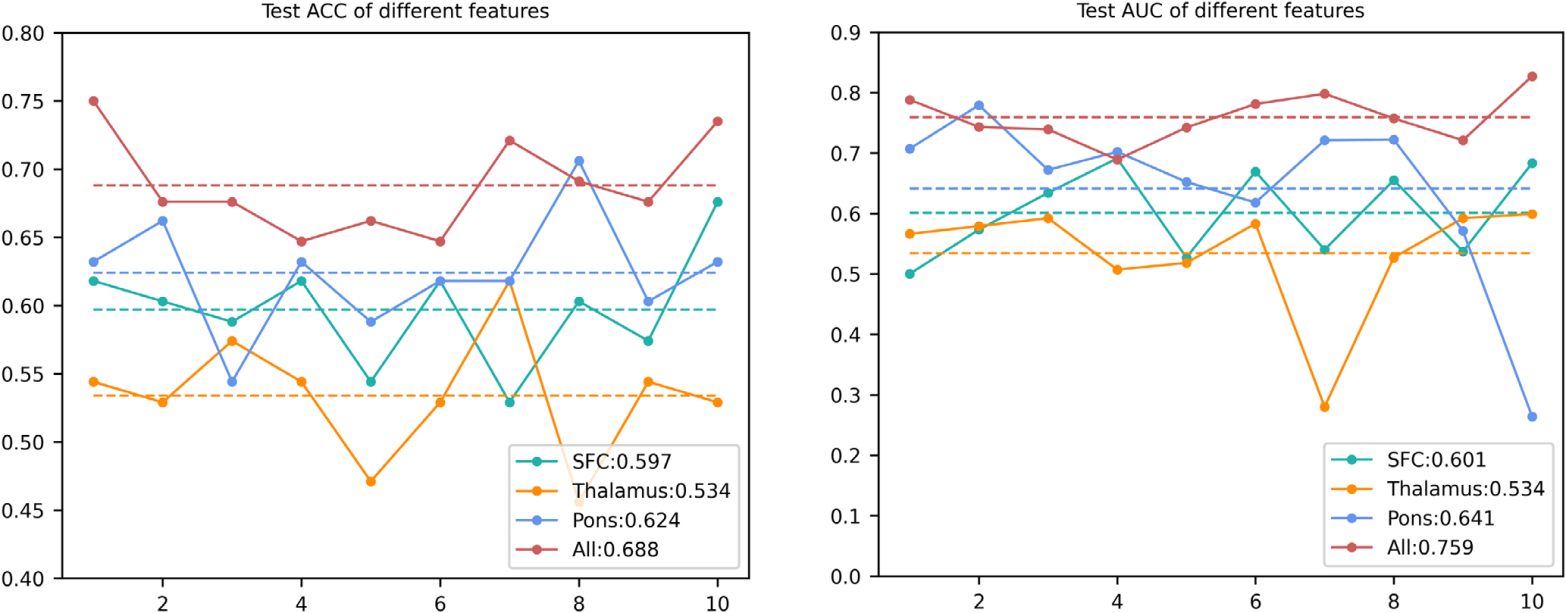
Machine learning model performance of different features. The accuracy (ACC) and area under the curve (AUC) of the three features in the test cohort are shown. The horizontal axis represents 10 splits, and the vertical axis represents the ACC and/or AUC values. The average ACC/AUC of an experiment is marked with dotted lines of the same colour as the broken line listed in the lower right corner. ACC, accuracy; AUC, area under the curve

**FIGURE 6:**
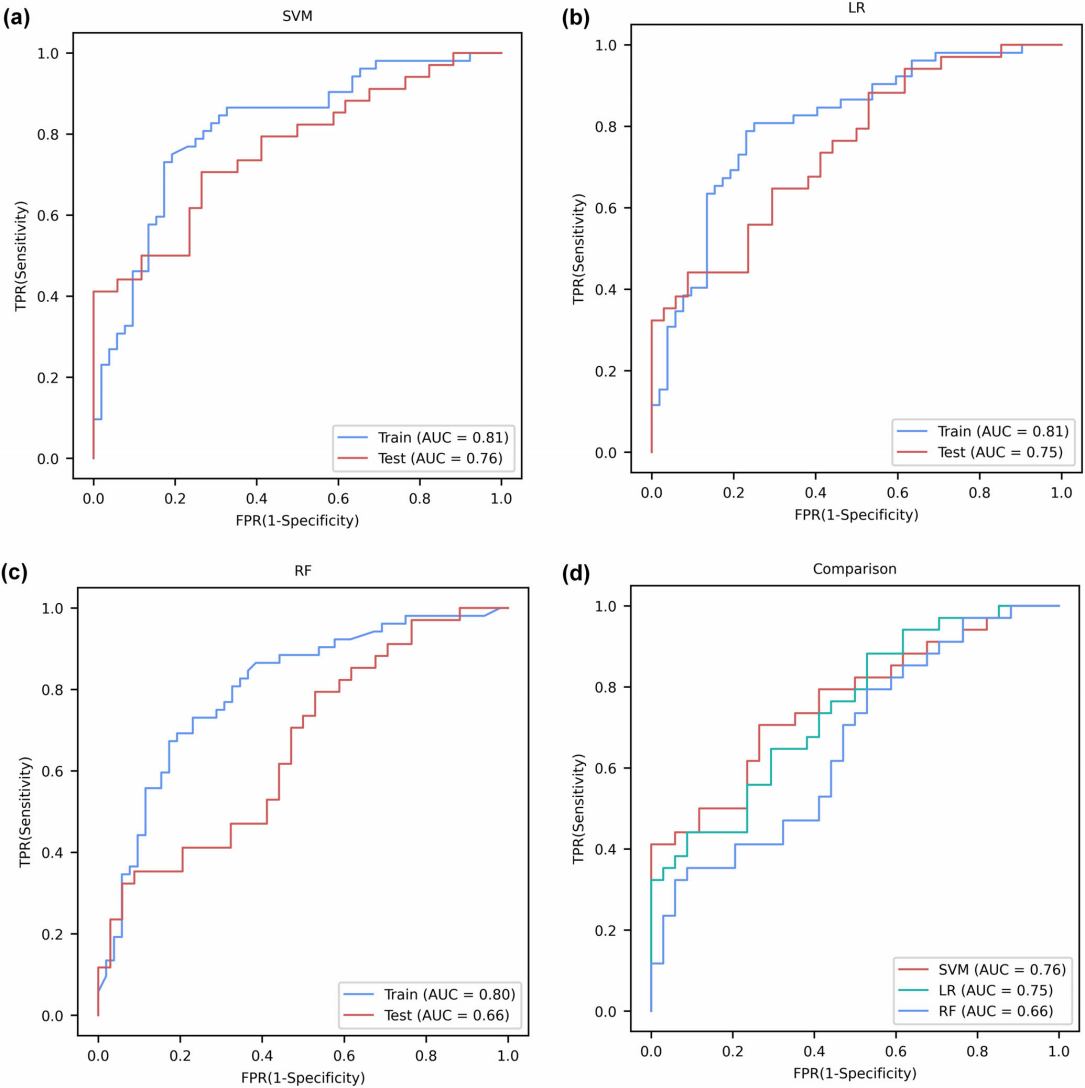
Receiver operating characteristic curves in machine learning. The receiver operating characteristic curves demonstrated the classification performance of combining the resting‐state functional connectivity of the habenula with all three brain regions by three machine learning methods. AUC, area under the curve; FPR, false‐positive rate; LR, linear regression; RF, random forest; SVM, support vector machine; TPR, true positive rate; train: the training set (*N* = 104); test, the test set (*N* = 68).

Using SVM learning, we achieved a classification accuracy of 75.9% in the training dataset and 68.8% in the test dataset based on a combination of the rsFCs of the habenula with three brain regions, including the SFC, thalamus, and pons, which was obviously superior to using the rsFC of the habenula with only one of these three regions (Figure 5). Using linear regression, we achieved a classification accuracy of 77.9% in the training dataset and 73.9% in the test dataset based on a combination of the rsFCs of the habenula with three brain regions. At last, using random forest, we achieved a classification accuracy of 73.1% in the training dataset and 55.9% in the test dataset based on a combination of the rsFCs of the habenula with three brain regions (Table S5). The model trained with a combination of all three features has a total *p* value of .001, .001, and .005 for SVM, linear regression and random forest, respectively (Table S5). The receiver operating characteristic curves of the three classifiers are shown in Figure 6.

### 3.7 Correlation analysis

The habenula‐SFC rsFC values were positively correlated with VAS (*r* = .434, *t* = 3.81, *p* < .001) and HAMD scores (*r* = .305, *t* = 2.59, *p* = .011) in patients with cLBP. The rsFC of the habenula‐right insula was negatively correlated with pain duration (*r* = −.366, *t* = −3.602, *p* = .001) in patients with cLBP. There were no significant correlations between habenular effective connectivity and pain characteristics/depression scores. Association analysis of habenular rsFC and effective connectivity suggested no significant correlations in patients with cLBP.

### 3.8 RsFC of the habenula with unsmoothed data

For the first dataset, patients with cLBP (52 subjects) showed significantly decreased rsFC of the habenula‐pons pathway, and enhanced rsFC of the habenula‐left SFC/right thalamus pathways as compared with HCs (52 subjects). For the second dataset, patients with cLBP (34 subjects) showed significantly enhanced rsFC of the habenula‐left SFC/right thalamus pathways, and decreased rsFC of the habenula‐periaqueductal grey (PAG) pathway as compared with HCs (34 subjects). The results are shown in the Supplemental Materials (Table S6, and Table S7).

## 4 DISCUSSION

Here, we compared the rsFC and effective connectivity of the habenula between cLBP patients and HCs and used these features to distinguish cLBP patients from HCs by machine learning methods. We found significantly different habenular connectivity patterns associated with pain intensities and/or depression states in patients with cLBP and HCs. SVM learning, linear regression, and random forest could reliably distinguish cLBP from HCs based on habenular rsFC.

CLBP is a multi‐dimensional experience including physiological, affective, cognitive, behavioural, and social components (Ho et al., 2022). The habenula is a small epithalamic structure which can process many functions like olfaction, ingestion, endocrine, reward function, and pain (Boulos et al., 2017). Recent research has implicated it in modulating pain and pain‐related behaviours such as depression and anxiety (Antunes et al., 2022; Dai et al., 2022). The habenula is divided into medial and lateral components associated with different neurochemical characteristics. The increased activity of the lateral habenula is thought to play a key role in pain‐depression comorbidity (Guang‐Fen et al., 2020). Enhancing the thalamic reticular nucleus‐lateral habenula circuit can relieve depression induced by chronic pain (Xu et al., 2023). Activation of the tachykinin receptor 3 in the lateral habenula may alleviate pain and anxiety in mice (W. W. Zhang et al., 2023). In this study, we did not differentiate the medial and lateral part of the habenula. Further study is necessary to explore the connectivity patterns of the medial and lateral respectively.

To start with, we found significantly enhanced rsFC of the habenula‐SFC pathway and decreased rsFC of the habenula‐pons pathway in cLBP patients compared with HCs. It has been suggested that the habenula receives inputs from forebrain structures and send efferents to brainstem regions, which are well‐known pain modulatory regions (Shelton, Becerra, & Borsook, 2012). Direct habenula‐prefrontal projections have been identified in humans by probabilistic tractography (Vadovicova, 2014). Reduced rsFC of the habenula‐prefrontal cortex has been reported in paediatric patients with complex regional pain syndrome (Erpelding et al., 2014) and those with neuropsychiatric disorders (Ely et al., 2016; Gan et al., 2018; Vadovicova, 2014), demonstrating its role in pain/emotion processing. The projections to the midbrain regions, including PAG and raphe nuclei, are important in habenula‐mediated analgesia (Behbehani, 1995) (Ren & Dubner, 2002; Wang & Nakai, 1994). We observed enhanced rsFC of the habenula‐SFC pathway and decreased rsFC of the habenula‐pons pathway in cLBP patients of the first cohort (52 cLBP vs. 52 HCs), as well as enhanced rsFC of the habenula‐MPF pathway and decreased rsFC of habenula‐pons pathway in the independent cohort (34 cLBP vs. 34 HCs). In addition, patients with cLBP showed significantly higher depression scores than HCs. These findings, together with the association of rsFC and pain characteristics/depression scores, might indicate altered pain and emotion processing by both afferents and efferents to the habenula in patients with cLBP.

Second, enhanced rsFC of the habenula‐thalamus and habenula‐insular (bilateral) pathways was also suggested in patients with cLBP compared with HCs. The rsFC of the habenula‐right insula was negatively associated with pain duration and HAMD scores. Thalamo–habenula connectivity has been thoroughly examined in various vertebrates but is not well defined in humans (Jesuthasan, 2018). Only a few studies have identified both structural and functional connectivity between the habenula and thalamus in humans by high/ultrahigh field MR (3.0 T–7 T) (Shelton, Pendse, et al., 2012; Strotmann et al., 2014; Torrisi et al., 2017). The connectivity difference in the thalamus in this study was located mainly in the anterolateral part of the thalamus, which was thought to have connectivity with the prefrontal cortex (Behrens et al., 2003; Hwang et al., 2017; Krauth et al., 2010). As a relay of noxious stimuli transmission from peripheral receptors to the cortex, the thalamus is thought to contain parallel ascending pathways (Groh et al., 2018), the lateral thalamocortical pathway coding the sensory discriminative aspects of pain, and the medial thalamocortical pathway coding the emotional qualities of pain (Groh et al., 2018). The connectivity difference of the bilateral insular cortex in this study lies mainly in the anterior parts. The insular cortex is part of the corticolimbic brain areas and has been consistently implicated in various acute and chronic pains (Thompson & Neugebauer, 2018). The decreased rsFC of the habenula‐right insular pathway might suggest an adaptation/maladaption to cLBP in such patients. Abnormal rsFC between the habenula and thalamus/insular cortices in cLBP may have a significant role in the chronification of LBP. Treatment targeting the habenula may be effective in the clinical management of cLBP.

Third, we performed a control seed analysis using the thalamus and found distinct abnormal rsFC of the habenula and thalamus. Patients with cLBP showed significantly abnormal rsFC between the thalamus and parietal/cingulate/insular cortices indicating abnormal thalamo‐motor or thalamo‐somatosensory connectivity in these patients. From the results, we can see that the thalamus‐based functional connectivity is obviously different from the habenula‐related functional connectivity. Patients with cLBP showed distinct rsFC of the habenula with regions of SFC, thalamus, insular cortex, and pons in cLBP patients. Which may largely reflect the reliability of our results of abnormal connectivity patterns of the habenula in cLBP patients.

For the independent cohort, patients with cLBP showed significantly enhanced rsFC of the habenula‐frontal cortex and habenula‐thalamus pathways, as well as decreased rsFC of habenula‐pons pathway when compared to their relative HCs. However, the pathways showing abnormal rsFC of the habenula were not entirely consistent with the first cohort. The inconsistency comes from the fact that enhanced rsFC of the habenula‐anterior insula pathway of cLBP was found only in the first cohort, and enhanced rsFC of the habenula‐posterior insula/left temporal cortex of cLBP was found only in the independent cohort. In addition, the specific location of the frontal cortex was not exactly the same for the two cohorts. LBP is a symptom accompanied by many pathoanatomical causes or with nonspecific causes (Maher et al., 2017). Previous studies have suggested “different pain, different brain” in that the thalamic volume decrease was only seen in the patients with trigeminal neuropathic pain but not in the patients with temporomandibular disorders (non‐neuropathic pain) (Gustin et al., 2011). Although the majority of CLBP was nonspecific, we did not differentiate the causes of cLBP in the two cohorts, which might have caused the inconsistency of the results. Both datasets suggested abnormal habenula rsFC with frontal cortex, thalamus and pons, indicating abnormal interactions between habenula and these brain regions in cLBP states.

DCM suggested significantly enhanced effective connectivity from the right thalamus to right habenula in cLBP patients compared with HCs in the first cohort and significantly enhanced effective connectivity of the pons‐to‐right habenula pathway in the independent cohort. There are several methods proposed to characterize effective connectivity for fMRI study, such as DCM (K. Friston, 2009), the analysis of Granger causality (Goebel et al., 2003), and structural equation modelling (Bavelier et al., 2000). Of them, DCM is a more consistent and informative approach to infer causal relationship between brain regions on the basis of fMRI data than others (David et al., 2008; K. Friston, 2009). Spectral DCM can be used to model not only task‐related effective connectivity (Kahan et al., 2014; Preller et al., 2019) but also resting‐state effective connectivity among coupled populations of neurons (K. J. Friston et al., 2014; Razi et al., 2015). Spectral DCM has been used to detect the effective connectivity within the default mode network in healthy subjects (Sharaev et al., 2016). The habenula has been considered a relay to transmit sensory information obtained from frontal areas (Shelton, Becerra, & Borsook, 2012) to the brainstem regions (Ellison, 1994). Our findings might suggest abnormal information transmission via the habenula in cLBP. Although the underlying mechanism remains largely unclear, the observed alteration in the effective connectivity of the habenula in the cLBP group might represent a maladaption of the information transmission driven by the habenula in such a chronic pain state.

In addition, we tried to test the relationship between rsFC and effective connectivity of the habenula but found no significant association between them in cLBP in the two cohorts. The rsFC and effective connectivity represent different things. RsFC represents endogenous fluctuations in BOLD signals throughout the brain (Fox & Greicius, 2010), while DCM values represent directional interactions among multiple brain areas (Frassle et al., 2021). Due to a lack of correlation, the potential mechanism remains obscure, and further research is necessary to elucidate the relationship between altered rsFC and effective connectivity in the pathogenesis of cLBP.

Machine learning is increasingly used in pain research to predict and assist diagnosis, successful decision‐making, and effective treatment of pain (Ichesco et al., 2021; Matsangidou et al., 2021). There are several machine learning algorithms including SVM, random forest, logistic regression, decision tree, and so forth that has been used to explore pain biomarkers (Boissoneault et al., 2017; Lotsch & Ultsch, 2018). Of them, SVM has been used in the discrimination and prediction of cLBP based on brain features such as cortical thickness and rsFC (Lamichhane et al., 2021; Shen et al., 2019; B. Zhang et al., 2019). In this study, three machine learning methods, that is, SVM, linear regression, and random forest, were used to classify cLBP patients and HCs based on the habenular rsFC features. We found that rsFC changes of the habenula with three brain regions (SFC, right thalamus, pons) could distinguish cLBP patients from HCs with equivalent accuracies of 75.9, 77.9, and 73.1%, respectively, by SVM, linear regression, and random forest. Which were validated in an independent cohort (accuracies = 68.8, 73.9, and 55.9%, respectively). Of the three methods, random forest obtained a classification accuracy of 55.9% in the test set which is not so good as SVM and linear regression. Generally speaking, our study validated that the habenular rsFC can be used as an imaging biomarker to distinguish cLBP patients from HCs.

Considering the smaller size of the habenula, 4 mm was selected as the FWHM Gaussian kernel in the spatial smoothing of the functional images in this study. In previous studies of the habenula, different smooth kernels have been used, including 5 mm (Shelton, Pendse, et al., 2012), 6 mm (Luan et al., 2019; L. Zhang et al., 2017), 2 mm (Lawson et al., 2014; Liu et al., 2017), 3 mm (Hetu et al., 2016), 4 mm (Qiao et al., 2020), and 8 mm (Gan et al., 2018). There is no consensus regarding the best smooth kernel. A smaller smooth kernel was thought to increase the signal‐to‐noise ratio without smoothing the signal beyond the limits of the habenula, as suggested by Lawson et al. (2014). However, in another study, larger voxels and larger smooth kernels were thought to improve sensitivity for detecting both cortical activation and hippocampal connectivity (Burman, 2021). Detecting the influence of different smooth kernels on habenular connectivity may be helpful to characterize habenular function most effectively. In future studies, we can compare the effects of different voxel sizes and smooth kernels on habenular connectivity to determine the optimal processing parameters and scanning parameters.

Habenula is a small structure and current fMRI techniques have limited spatial resolution, a previous study has suggested to do rsFC analysis for small brain structure without smoothing the fMRI data (Mills et al., 2018). We repeated the rsFC analysis with unsmoothed data. The results are largely consistent with those obtained from smoothed data. While there are some differences for the results from the smoothed and unsmoothed data. First, no significant between‐group differences of the habenula‐bilateral insular cortex were found between 52 cLBP patients and 52 HCs with unsmoothed data. Second, no significant between‐group difference of the rsFC of the habenula‐pons pathway was suggested between 34 cLBP patients and 34 HCs with unsmoothed data but the difference lied in the habenula‐PAG pathway in these unsmoothed data. There is no consensus about whether to smooth, or the selection of smooth kernel in fMRI study of different brain structures. It is unknown whether unsmoothing can reduce the sensitivity of detecting the habenula connectivity. It will be helpful to test the effect of smoothing/unsmoothing on the detection of rsFC for small brain structures in future studies.

There were several limitations. First, the habenula consists of the medial and lateral parts, but we did not divide it into two parts in MRI images due to the smaller size. Second, the results of the study should be interpreted cautiously because we only reported the functional characteristics of the habenula in cLBP. The structural properties should be studied in future research, and the match between structure and function would be more helpful to understand its mechanism in chronic pain. High‐resolution MR scans (e.g., 0.3–0.7 mm) by ultrafield MR or more precise segmentation techniques might help to clarify the structural changes of the habenula in patients with cLBP (Lawson et al., 2013; Torrisi et al., 2017). Third, this is a cross‐sectional study, which precludes us from making causal inferences about the relationship between altered habenular connectivity and clinical symptoms.

## 5 CONCLUSION

In conclusion, we observed altered rsFC and effective connectivity of the habenula associated with pain characteristics/depression states in patients with cLBP. Machine learning can be used to classify cLBP patients and HCs based on the habenular rsFC. Our study highlights the abnormality of the rsFC and effective connectivity of the habenula in cLBP and the promise of machine learning in chronic pain discrimination. Future studies with longitudinal designs may help to elucidate the functional importance of the habenula in cLBP.

## CONFLICT OF INTEREST STATEMENT

The authors declare no conflict of interest.

## DISCLOSURE

The data collection and sharing for this project in the independent cohort was provided by the OpenPain Project (OPP; Principal Investigator: A. Vania Apkarian). OPP funding was provided by the National Institute of Neurological Disorders and Stroke (NINDS) and National Institute of Drug Abuse (NIDA). OPP data are disseminated by the Apkarian Lab, Physiology Department at the Northwestern University, Chicago.

## Supporting information


**FIGURE S1** Spherical regions of interest in bilateral thalamus.
**FIGURE S2** Between‐group comparisons of the thalamus‐cortical functional connectivity between 52 healthy controls and 52 patients with chronic low back pain.
**TABLE S1** Group differences of the resting‐state functional connectivity of the thalamus between 52 healthy controls and 52 patients with chronic low back pain.
**TABLE S2** Demographical and clinical data of the independent cohort.
**TABLE S3** Group differences of the resting‐state functional connectivity of the habenula in the independent cohort.
**TABLE S4** Mean connection strengths (in Hz) of 34 healthy controls and 34 patients with chronic low back pain in the independent cohort.
**TABLE S5** Classification performance of the three classifiers
**TABLE S6** Group differences in the resting‐state functional connectivity of the habenula with unsmoothed data between 52 cLBP patients and 52 healthy controls
**TABLE S7** Group differences in the resting‐state functional connectivity of the habenula between 34 cLBP patients and 32 healthy controls.

## Data Availability

The data of the 104 participants are in‐house dataset and are available from the corresponding author upon reasonable request. The data in the independent cohort can be freely obtained from the website “openpain.org” provided by OpenPain Project https://openpain.org/html/agreement.html (OPP; Principal Investigator: A. Vania Apkarian).
