# Supplementary material for: Altered habenular connectivity in chronic low back pain: An fMRI and machine learning study
Source: Hum Brain Mapp. 2023 Jun 12;44(11):4407–21. doi: 10.1002/hbm.26389 (PMC10318213; doi:10.1002/hbm.26389)
Supplement: Supplementary file 1 — FIGURE S1 Spherical regions of interest in bilateral thalamus. FIGURE S2 Between‐group comparisons of the thalamus‐cortical functional connectivity between 52 healthy controls and 52 patients with chronic low back pain. TABLE S1 Group differences of the resting‐state functional connectivity of the thalamus between 52 healthy controls and 52 patients with chronic low back pain. TABLE S2 Demographical and clinical data of the independent cohort. TABLE S3 Group differences of the resting‐state functional connectivity of the habenula in the independent cohort. TABLE S4 Mean connection strengths (in Hz) of 34 healthy controls and 34 patients with chronic low back pain in the independent cohort. TABLE S5 Classification performance of the three classifiers TABLE S6 Group differences in the resting‐state functional connectivity of the habenula with unsmoothed data between 52 cLBP patients and 52 healthy controls TABLE S7 Group differences in the resting‐state functional connectivity of the habenula between 34 cLBP patients and 32 healthy controls. [file HBM-44-4407-s001.doc]

**Supplemental materials**

**Title:** Altered habenular connectivity in chronic low back pain: a fMRI and machine learning study

1. **Medications for 52 patients with chronic low back pain**

The medications for all patients with chronic low back pain were recorded. Fifteen patients were untreated. Thirteen patients took oral painkillers (i.e., Nonsteroidal Anti-inflammatory drugs, or drugs removing the phlegm and turbid urine). Nine patients were treated with oral Chinese herbal medicine or plaster. Three patients took placebo. Ten patients selected physiotherapy, acupuncture, or lumbar traction therapy. Two patients modified their lifestyle (regular exercise).

1. **Control seeds of thalamus and the connectivity analysis results based on the control seeds.**


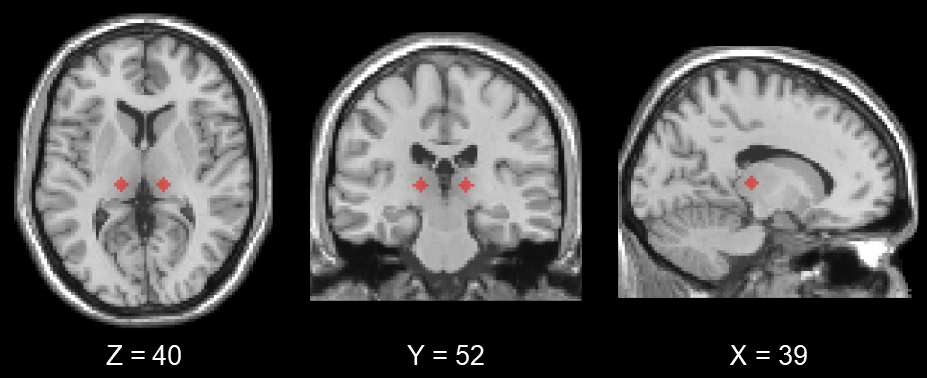


Figure S1 Spherical regions of interest (radius = 4mm) in bilateral thalamus.


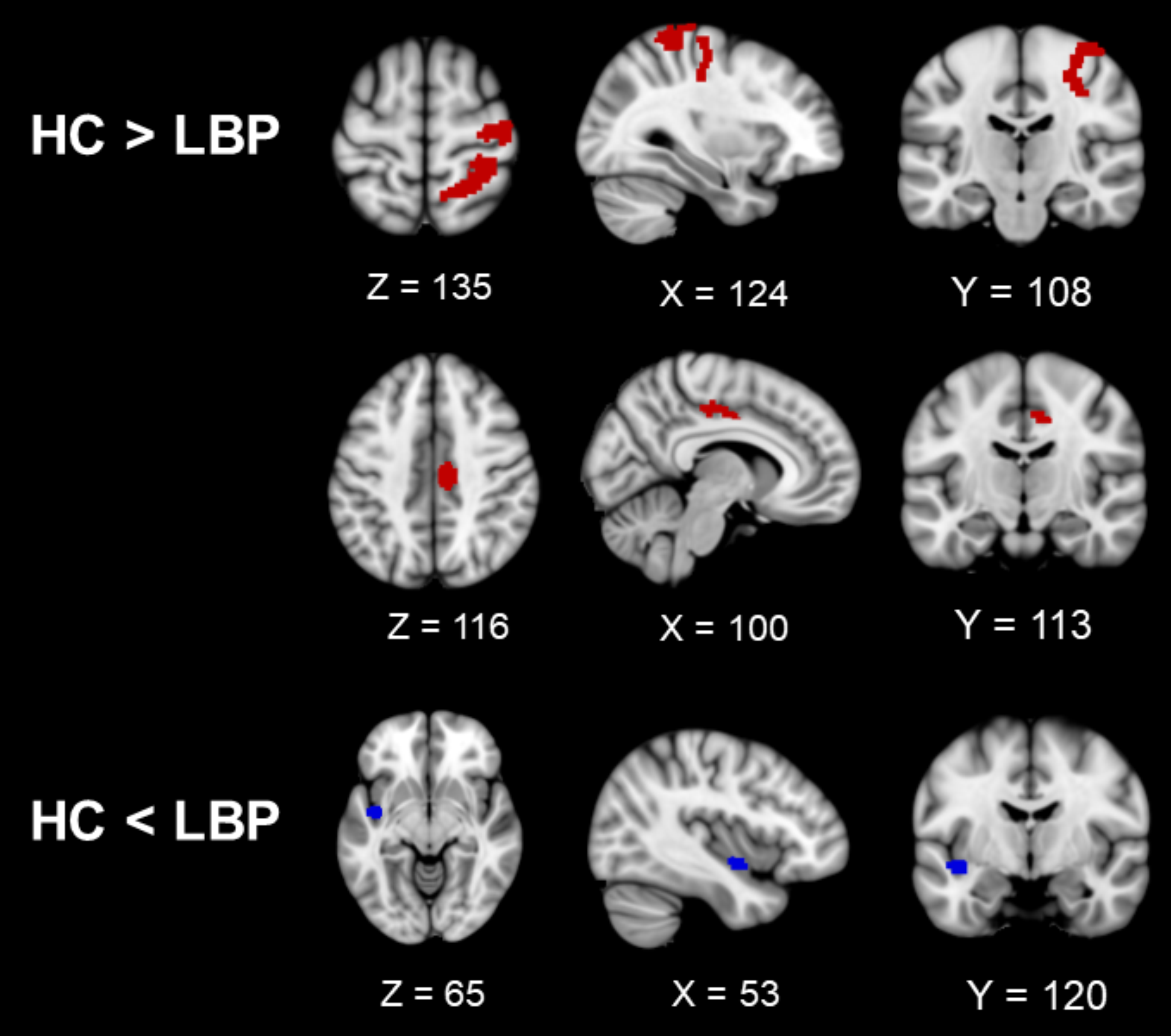


Figure S2 Between-group comparisons of the thalamus-cortical functional connectivity. blue: controls below than patients; red: controls above than patients. HC, healthy controls; LBP, low back pain.

Table S1 Group differences of the resting-state functional connectivity of the thalamus.

| Group difference | Target area | Volume data | | | Voxels | Z value | *p* |
| --- | --- | --- | --- | --- | --- | --- | --- |
|  |  | X | Y | Z |  |  |  |
| HC < CLBP | Left posterior insular cortex | -40 | -4 | 10 | 93 | 4.21 | < 0.01 |
| HC > CLBP | Right parietal cortex, right precentral cortex | 30 | -38 | 68 | 830 | 4.18 | <0.001 |
|  | Cingulate cortex | 12 | -20 | 46 | 155 | 4.0 | 0.01 |

HC, healthy control; CLBP, chronic low back pain; MPFC, medial prefrontal cortex; PAG, periaqueductal gray.

**3. The demographical material of the independent cohort.**

**Table S2 Demographical and** clinical data of the independent cohort.

| Item | HC | CLBP | *P* |
| --- | --- | --- | --- |
| Number of all subjects | 34 | 34 |  |
| Number of females (%) | 15(44%) | 15 (44%) | 0.941 |
| Age (years) | 49.3 ± 9.1 ^a^ | 49.2 ± 8.6 | 0.967 |
| Age range (years) | 21 – 64 | 21 – 62 |  |
| BDI | 1.5 ± 2.6 | 6.3 ± 5.8 | ＜ 0.001 |
| VAS score | 0 | 6.7 ± 1.7 | NA |
| Disease duration (years) | 0 | 15.7 ± 11.3 | NA |

HC, healthy control; CLBP, chronic low back pain; BDI, Beck Depression Index; VAS, visual analogue scale; NA, not available.

^a^: mean ± standard deviation.

**4. Results of connectivity analysis of the independent cohort.**

**4.1 Results from resting-state functional connectivity of the habenula.**

**Table S3 Group differences of the resting-state functional connectivity of the habenula in the independent cohort.**

| Group difference | Target area | Volume data | | | Voxels | Z value | *p* |
| --- | --- | --- | --- | --- | --- | --- | --- |
|  |  | X | Y | Z |  |  |  |
| **HC** < CLBP | Right MPFC | 6 | 24 | 40 | 158 | 4.27 | 0.003 |
|  | Right thalamus | 12 | -12 | 12 | 10 | 3.19 | 0.015 |
|  | left temporal cortex | -48 | -60 | -4 | 214 | 4.14 | ＜0.001 |
| **HC** > CLBP | Pons | 12 | -28 | -40 | 12 | 3.44 | 0.012 |

HC, healthy control; CLBP, chronic low back pain; MPFC, medial prefrontal cortex; PAG, periaqueductal gray.

**4.2 DCM analysis for the independent cohort**

There were 5 regions included in the DCM analysis and the MNI coordinates of the region’s centers were as follows: the MPF (x = 6, y = 12, z = 40), thalamus (x = 14, y = -12, z = 12), left habenula (x = -3, y = -24, z = 2), right habenula (x = 5, y = -24, z = 2) and PAG (x = 2, y = -36, z = -14). DCM analysis revealed that the fully connected model was the best model in both the cLBP and healthy controls. It was the best model for 29 of 34 healthy controls, and for 29 of 34 cLBP patients. For healthy controls, model 2 was the best model for 5 subjects. For patients with cLBP, model 2 and model 3 were the best model for 3 and 2 subjects respectively.

**4.3 Results from the DCM analysis.**

**Table S4 Mean connection strengths (in Hz) of 34 healthy controls and 34 patients with chronic low back pain in the independent cohort.**

| Group | BMS | From SFC | From Tha | From LHb | From RHb | From Pon |
| --- | --- | --- | --- | --- | --- | --- |
| **HC** | To SFC | 0 | 0.09* | 0.07* | 0.034 | 0.084* |
|  | To Tha | -0.109 | 0 | -0.002 | 0.032 | -0.033 |
|  | To LHb | -0.427** | -0.25** | 0 | 0.349** | -0.551** |
|  | To RHb | -0.358** | -0.204** | 0.068 | 0 | -0.528** |
|  | To Pon | -0.045 | -0.051 | 0.002 | 0.058 | 0 |
| **CLBP** | To SFC | 0 | 0.061 | 0.054* | 0.07** | 0.052 |
|  | To Tha | -0.112* | 0 | 0.055 | -0.015 | -0.033 |
|  | To LHb | -0.433** | 0.003 | 0 | 0.214* | -0.329** |
|  | To RHb | -0.363** | -0.072* | 0.271** | 0 | -0.223*△ |
|  | To Pon | -0.031 | 0.051 | 0.036 | 0.051 | 0 |

There are source regions in rows and target regions in columns. HC, healthy control; CLBP, chronic low back pain; BMS, Bayesian Model Selection; SFC: superior frontal cortex; Tha, thalamus; LHb, left habenula; RHb: right habenula. *: *P* < 0.05; **, *P* < 0.01. The “△” represents the results from between-group comparisons of the habenular effective connectivity.

**5. Results from machine learning.**

**Table S5 Classification performance of the** three classifiers

| **Classifier** | **Cross score** | **Training set** | | | | | **Testing set** | | | | | |
| --- | --- | --- | --- | --- | --- | --- | --- | --- | --- | --- | --- | --- |
|  |  | **ACC** | **AUC** | **Pre** | **Sen** | **Spe** | **ACC** | ***P-value*** | **AUC** | **Pre** | **Sen** | **Spe** |
| **SVM** | 0.739 | 0.759 | 0.812 | 0.737 | 0.808 | 0.712 | 0.688 | 0.001 | 0.759 | 0.7 | 0.617 | 0.735 |
| **LR** | 0.739 | 0.779 | 0.808 | 0.773 | 0.788 | 0.769 | 0.739 | 0.001 | 0.752 | 0.747 | 0.677 | 0.617 |
| **RF** | 0.72 | 0.731 | 0.8 | 0.714 | 0.769 | 0.692 | 0.559 | 0.005 | 0.66 | 0.559 | 0.559 | 0.559 |

SVM, support vector machine; LR, linear regression; RF, random forest; ACC, accuracy; AUC, area under curve; Pre, precision; Spe, specificity; Sen, sensitivity.

6. Results of the habenular resting-state functional connectivity of the patients with chronic low back pain as compared with healthy controls with unsmoothed fMRI data.

**6. Results from resting-state functional connectivity analysis with unsmoothed data.**

**Table S6 Group differences in the resting-state functional connectivity of the habenula** between 52 cLBP patients and 52 healthy controls

| Group difference | Target area | Volume data | | | Voxels | Z value | *P* |
| --- | --- | --- | --- | --- | --- | --- | --- |
|  |  | X | Y | Z |  |  |  |
| HC < CLBP | Left SFC | -10 | 6 | 56 | 126 | 3.65 | 0.011 |
|  | Right thalamus | 22 | -14 | 14 | 12 | 3.69 | 0.019 |
| HC > CLBP | Pons | 2 | -18 | -32 | 48 | 4.37 | 0.006 |

HC, healthy control; CLBP, chronic low back pain; SFC, superior frontal cortex. The *p* values were corrected for false discovery rate.

**Table S7 Group differences in the resting-state functional connectivity of the habenula between 34 cLBP patients and 32 healthy controls**.

| Group difference | Target area | Volume data | | | Voxels | Z value | *P* |
| --- | --- | --- | --- | --- | --- | --- | --- |
|  |  | X | Y | Z |  |  |  |
| HC < CLBP | Left SFC | -12 | 12 | 40 | 138 | 4.9 | 0.003 |
|  | Right thalamus | 12 | -14 | 10 | 47 | 4.07 | 0.009 |
| HC > CLBP | PAG | 2 | -36 | -12 | 14 | 4.78 | 0.006 |

HC, healthy control; CLBP, chronic low back pain; SFC, superior frontal cortex. The *p* values are corrected for false discovery rate.
